# Modality, presentation, domain and training effects in statistical learning

**DOI:** 10.1038/s41598-022-24951-7

**Published:** 2022-12-03

**Authors:** Krisztina Sára Lukics, Ágnes Lukács

**Affiliations:** 1 Department of Cognitive Science, Budapest University of Technology and Economics, Műegyetem rkp. 3., H-1111 Budapest, Hungary; 2 MTA-BME Momentum Language Acquisition Research Group, Eötvös Loránd Research Network (ELKH), Budapest, Hungary

**Keywords:** Psychology, Human behaviour

## Abstract

While several studies suggest that the nature and properties of the input have significant effects on statistical learning, they have rarely been investigated systematically. In order to understand how input characteristics and their interactions impact statistical learning, we explored the effects of modality (auditory vs. visual), presentation type (serial vs. simultaneous), domain (linguistic vs. non-linguistic), and training type (random, starting small, starting big) on artificial grammar learning in young adults (N = 360). With serial presentation of stimuli, learning was more effective in the auditory than in the visual modality. However, with simultaneous presentation of visual and serial presentation of auditory stimuli, the modality effect was not present. We found a significant domain effect as well: a linguistic advantage over nonlinguistic material, which was driven by the domain effect in the auditory modality. Overall, the auditory linguistic condition had an advantage over other modality-domain types. Training types did not have any overall effect on learning; starting big enhanced performance only in the case of serial visual presentation. These results show that input characteristics such as modality, presentation type, domain and training type influence statistical learning, and suggest that their effects are also dependent on the specific stimuli and structure to be learned.

## Introduction

Our surroundings are full of structured patterns and regularities. In order to efficiently operate in this complex environment, an organism has to be equipped with abilities to find, learn, and utilize these environmental structures and regularities. Statistical learning is a powerful mechanism of extracting and encoding structure from environmental stimuli^1^. This form of learning is ubiquitous in human cognition: studies have shown that it is present in the auditory, visual, and tactile modalities and across the linguistic and nonlinguistic domains^2–12^, and it also operates in multimodal, visuomotor tasks^13,14^, as well.

Statistical learning supports many skills in our everyday life. For instance, language, consisting of complex patterns and regularities on multiple levels, has been suggested to rely on it^15–25^. While the contribution of statistical learning might be most frequently highlighted in language, several results have shown that this mechanism is not limited to it: it has an important role in domains such as music acquisition^26^, event processing^27^, or acquiring complex visual stimuli like scenes or faces^28^, suggesting a diverse and varied role for statistical learning in human cognition. As the human cognitive system faces great diversity in learning materials, differences in the properties of the input may impose different constraints on statistical learning in each area^1,29^. Input constraints are especially important in statistical learning because this form of learning is model-free and input-driven compared to other forms of learning like reinforcement learning or declarative learning^29^. To understand how this fundamental mechanism operates in different areas of cognition, we aim to uncover how input characteristics and their interactions affect learning.

While the variability of areas where statistical learning is present may suggest generality, direct comparisons of learning the same structure with stimuli from different domains and modalities indicate the presence of modality and domain specific constraints. (In the present paper, we use *domain* to refer to the content of representations, more specifically, to denote the linguistic-nonlinguistic distinction in our tasks). These effects have mostly been demonstrated in artificial language learning tasks, where a few novel items are organized into sequences based on simple patterns. After being exposed to a set of grammatical sequences, humans are able to distinguish grammatical from ungrammatical sequences. These studies have shown that the efficiency of statistical learning of serially presented (i.e., presenting one stimulus after the other) nonlinguistic auditory patterns exceeds the extraction of serial nonlinguistic visual patterns, which in turn is better than learning serial nonlinguistic tactile patterns^3^. In general, statistical learning is assumed to have modality- or even stimulus specific characteristics^1,29^.

Importantly, these modality effects are likely to result from differences in parameters of optimal presentation. While sequential information in the auditory modality is only available through serial presentation of stimuli, for visual sequences, serial and simultaneous presentation are both feasible. Simultaneous presentation, where items of a sequence are presented together at the same time, seems to be the optimal in the case of statistical learning of nonlinguistic visual sequences^30,31^. When visual information is presented simultaneously, performance is similar to nonlinguistic auditory learning^30,31^. Presentation rates also affect learning differently in different modalities, and slower rates seem to facilitate visual statistical learning: when presentation rate is slower with serial linguistic visual than with serial linguistic auditory stimuli, learning performance is equivalent between the modalities^69^.

While modality differences in statistical learning have been demonstrated in several studies, tests of domain effects, e.g. direct comparisons of linguistic versus nonlinguistic materials are hard to find. One notable exception is Saffran^31^, who explored both domain (linguistic versus nonlinguistic) and modality (auditory versus visual) effects in an artificial grammar learning task and found no overall advantage of sequence learning in the linguistic over the nonlinguistic domain (or in the auditory over visual modality) with serial presentation of sequences. However, the focus of this study was on contrasting two types of grammars (grammars with predictive and non-predictive dependencies) within each condition, instead of directly comparing performance across domains and modalities. Although it was not the primary focus of their studies, Hoch, Tyler and Tillmann^70^ directly compared statistical learning in the linguistic and nonlinguistic domains, observing significantly higher levels of learning in the linguistic than in the nonlinguistic domain.

Besides constraints by modality, presentation type and domain, different arrangements of stimuli during training (training type) also influence statistical learning. The *starting small* hypothesis assumes that incremental presentation of stimuli of different length (and complexity) enhances statistical learning in the case of humans and neural networks^32^. In another formulation, under the *less is more* hypothesis^33,34^, cognitive limitations like reduced working memory capacity, help learning complex patterns and systems. Research on human learners and *less is more*/*starting small* is methodologically diverse and has given controversial results^35–38^. On the one hand, contrary to the predictions of the *less is more* hypothesis, several studies found that the acquisition of grammar structures in artificial grammar learning tasks is more effective in adults than in children^39^. On the other hand, simulating reduced working memory capacity in adults seems to facilitate learning in some^40^, but not in other studies^41^. *Starting big* arrangement of stimuli, starting with the longer strings of the grammar, has also been argued and demonstrated to result in superior performance by allowing larger chunks to be learned first and be parsed later^42^. However, it may also lead to false hypotheses about grammar structure^32,43^, or prevent generalization of rules^40^. To summarize, *starting small* and *starting big* training types lead to more efficient learning in some cases, but further research is needed to test the conditions in which they boost learning.

**Figure 1:**
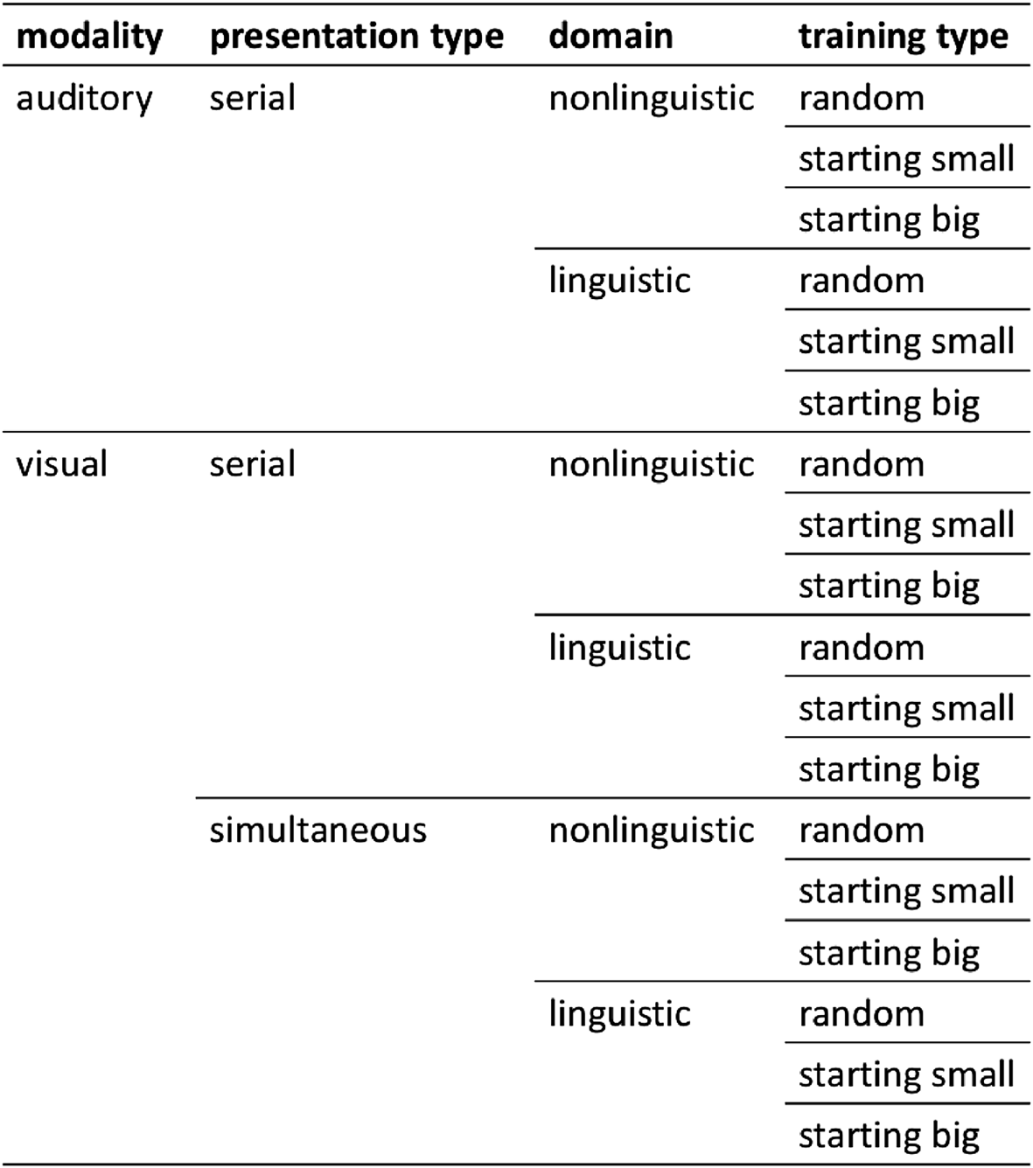
The design of the study. We systematically investigated the effect of four factors: modality (*auditory vs. visual*), presentation type (*serial* vs. *simultaneous*), domain (*linguistic* vs. *nonlinguistic*), and training type (*random* vs. *starting small* vs. *starting big*), yielding 18 conditions altogether. With 20 participants in each group, 360 participants took part in the study.

Although effects of input characteristics like modality, presentation, domain and training type have been examined before, previous research only investigated these effects on statistical learning separately calling for further studies with direct comparisons. Furthermore, many studies used different statistical learning designs, with differences in patterns, stimuli and presentation arrangement. Our aims in this study were to examine modality, presentation, domain and training type effects using Saffran’s^31^ predictive grammar in order to extend the results of the original study (a) by systematically investigating all combinations of the examined input effects and their interactions (e.g. by also including visual linguistic conditions), and (b) by directly comparing learning performance across conditions. We compare the efficiency of statistical learning in *visual* (both in *serial* and *simultaneous* presentation types) and auditory modalities and across *linguistic* versus *non-linguistic* domains. We also wanted to test how training type, namely *starting small* and *starting big* influences learning across these conditions, as training effects have not been examined with finite state, category-based grammars. Figure 1 summarizes the design of experimental conditions in the study.

Our hypotheses were the following:

1. Based on previous findings, we expected the advantage of learning in the auditory modality over the visual modality with serial presentation. We also hypothesized that when presentation is optimized for modality, this advantage disappears, and performance in the serial auditory and in the simultaneous visual tasks would be on similar levels.
2. In the present study, we aimed at directly comparing the acquisition of statistical patterns in the linguistic and nonlinguistic domains. Based on the results of Saffran^31^, we expected that the linguistic versus nonlinguistic status of stimuli would not have an effect on learning efficiency.
3. Training effects, starting small and starting big, have not been examined with finite state, category-based grammars. We hypothesized that starting small would facilitate learning compared to presenting training sequences in a random order, as starting small enables the generation of simple and flexible hypotheses about the rule^32,43^. In contrast, starting big would mainly facilitate learning of specific item-relations, and as a result, we expected it would result in lower learning performance than random training due to less effective hypothesis generation^42^.

These hypotheses can be translated to the following formulations in our current experimental design, motivating three sets of analyses:

1. With serial presentation of stimuli, we expected the advantage of learning in the auditory modality in comparison to the visual modality. We hypothesized that there would be no domain effect, that is, the linguistic conditions would not differ from the nonlinguistic conditions. We also expected that starting small training would lead to higher, while starting big would lead to lower performance than presenting training sequences with a different length in a random order.
2. In the visual conditions, we expected the advantage of simultaneous over serial presentation. In this case, we also expected no domain effect, and an advantage of starting small and disadvantage of starting big stimuli relative to random training.
3. With presentation optimized for modality, we expected that performance in the serial auditory and in the simultaneous visual tasks would be on similar levels. Here, we also hypothesized no domain effect, and an advantage of starting small and disadvantage of starting big stimuli relative to random training.

## Method

### Participants

360 young adults participated in the study. Most of them were university students who were recruited through facultative cognitive psychology courses at the Budapest University of Technology and Economics, and received course credit for their participation. The rest of the participants were volunteers who were recruited via convenience sampling. Inclusion criteria were normal or corrected-to-normal hearing and vision, and Hungarian as a native language. Participants were asked to report any neurological and psychiatric conditions (none were reported in our sample). Mean age was 22.5 (SD = 3.9, minimum = 18.1, maximum = 55.8), and 255 females and 105 males participated in the study. Age information was missing in the case of two participants. All participants were tested with their informed consent, in accordance with the principles set out in the Declaration of Helsinki and the stipulations of the local institutional review board (United Ethical Review Committee for Research in Psychology, ethical approval number: EPKEB-2018/87).

### Stimuli

**Figure 2:**
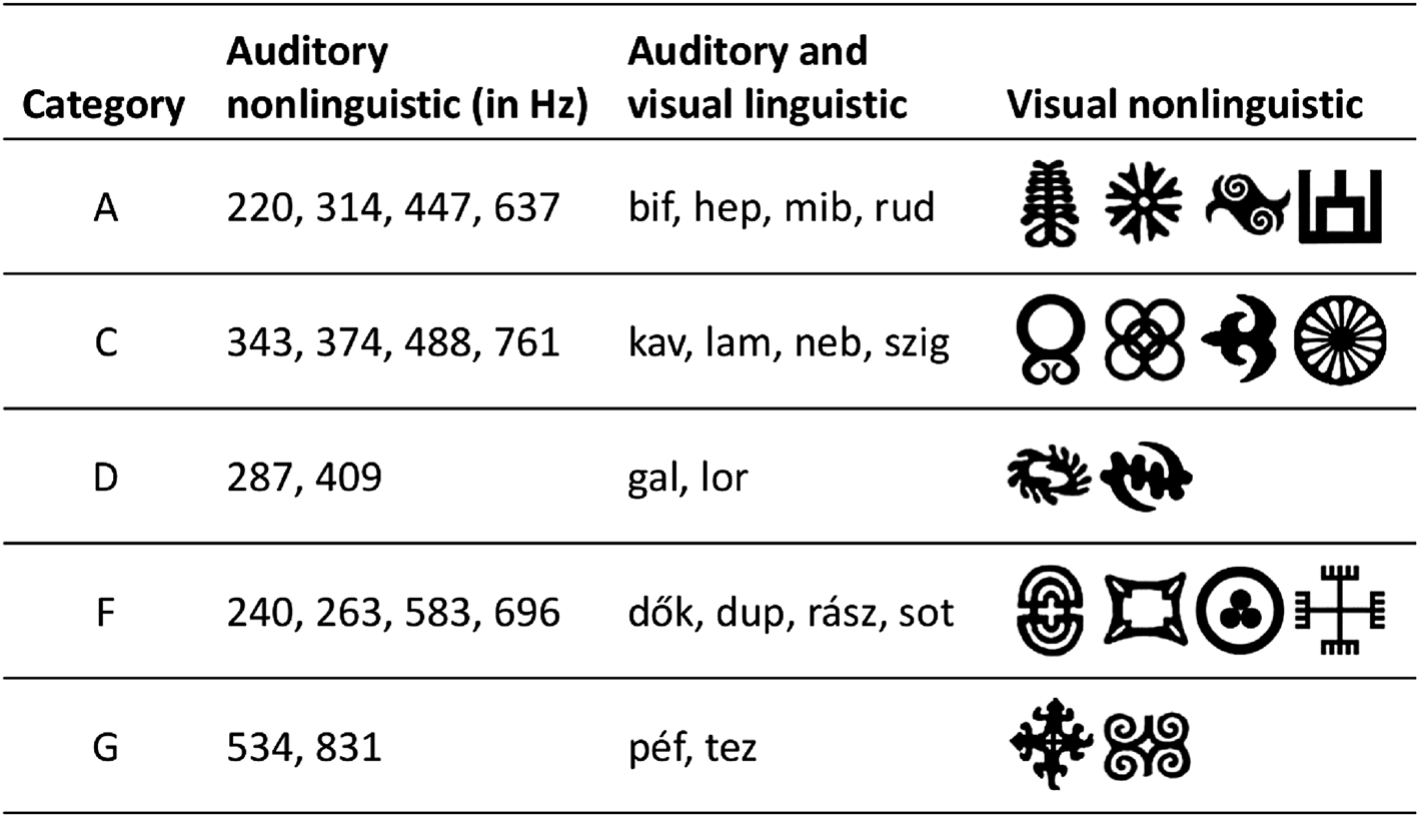
Stimulus sets in different conditions by modality and domain. Stimuli in each condition are classified into categories (A, C, D, F, and G). The rules of the grammar are defined on these categories.

Throughout the conditions, stimuli varied by modality (*auditory* versus *visual*) and domain (*nonlinguistic* versus *linguistic*). For all conditions, our aim was to design diverse stimulus sets where individual stimuli are well discriminable from each other. For the *auditory nonlinguistic* conditions, we divided a frequency range that was conveniently perceivable through our laboratory headphones (220–831 Hz) into 15 equal sections following steps of the musical scale to obtain 16 tones. As a result, we obtained intervals larger than standard semitones, and almost as large as standard whole tones (220 Hz, 240 Hz, 263 Hz, 287 Hz, 314 Hz, 343 Hz, 374 Hz, 409 Hz, 447 Hz, 488 Hz, 534 Hz, 583 Hz, 637 Hz, 696 Hz, 761 Hz, 831 Hz). Each tone was 470 ms long. For the *auditory linguistic* conditions, we used Hungarian CVC nonwords compiled from diverse Hungarian phonemes to promote discriminability (bif, dők, dup, gal, hep, kav, lam, lor, mib, neb, péf, rász, rud, szig, tez, sot). Note that some of the nonwords are four characters long as they include phonemes with a digraph (two-character grapheme) equivalent (‘sz’). Nonwords were recorded from a Hungarian female speaker, and the average length of syllables was 470 ms. In the *visual nonlinguistic* condition, 16 meaningless symbols were used that were rich in detail, and easily distinguishable from each other. In the *visual linguistic* conditions, the same syllables were used as in the auditory linguistic condition. Syllables were visually presented on white screen in black font. Individual items in each stimulus type were assigned to categories (as illustrated in Fig. 2), and the rules of the artificial grammar were defined over these categories.

**Figure 3:**
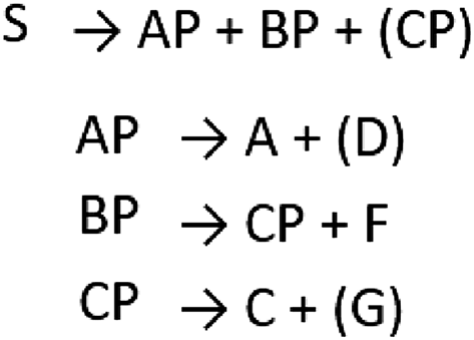
Rules of the artificial grammar (from Saffran^31^). Letters *A, C, D, F* and *G* refer to categories, which each include a set of items (tones, syllables, or symbols in the different conditions). Items in each category were randomly distributed in sentences. A sentence consists of an “AP” phrase, a “BP” phrase, and an optional “CP” phrase. An “AP” phrase is made of an “A” category item and an optional “D” category item. A “CP” phrase consists of a “C” and an optional “G” item. A “BP” phrase is made of a “CP” phrase and an optional “F” item.

With the help of the grammar (given in Fig. 3, taken from Saffran^31^) and the condition-specific categorized vocabularies, we generated 58 three to five items long grammatical sentences and 32 two to three items long phrases for the learning phases (90 sequences altogether). Phrases were parts of grammatical sentences. We also generated 24 pairs of grammatical and ungrammatical sequences (9 four- and 15 five item long sequences) for the test phases in all conditions. Grammatical sentences followed the rules of the grammar, while the ungrammatical ones included a violation of one of the grammatical rules: (1) sentences must contain an AP phrase, (2) D words follow A words, while G words follow C words, (3) sentences must contain an F word, (4) CP phrases must precede F words. As a result, there were four violation types, one for each rule: (1) sentences starting with a BP phrase instead of an AP phrase, (2) sentences where D and G words were interchanged so G words followed A words and D words followed C words, (3) sentences where F words were exchanged for G words, (4) sentences where CP phrases or parts of the CP phrases were missing before F words. Each violation type was represented by six ungrammatical strings. Members of categories were randomly distributed in sentences in the case of each category. The full set of training and test sequences together with their statistical properties for the linguistic conditions are included as supplementary material online. Sequences of the nonlinguistic conditions were parallel to those of linguistic conditions, that is, each syllable corresponded to a pure tone and a symbol, respectively. The modality and domain variations on conditions only differed in their stimulus set.

### Procedure

Participants were tested in a silent room in groups of two or three. The testing was administered using E-Prime 2.0 Professional. The test administration took cca. 15 min, and consisted of a training phase and a test phase for all conditions.

In the *auditory* conditions, items were presented with no pauses between them. In the *visual* conditions, we applied two presentation types: in the *serial* conditions, one item was presented at a time on the center of the screen for 800 ms, followed by the next item with no pauses, while in the *simultaneous* conditions, all items of a sentence were presented together on the screen at the same time. (Pilot data from our lab on a simpler segmentation task showed no learning effect in the case of visual statistical learning when stimulus timing was matched to that of acoustic statistical learning and set to 470 ms. This was one of the reasons for using a longer presentation time: we wanted to avoid floor effects in a more complex task in the visual modality. Choosing longer presentation times was also motivated by earlier studies showing that longer presentation times in visual statistical learning indeed promote learning^69,76,77^. Since visual presentation rates vary between 400 and 1200 ms in the literature, we decided to go with a mid-range 800 ms that was significantly longer than what we used in our pilot studies.) Presentation time was adjusted to sequence length (the number of items times 800 ms). During the training phase, participants were instructed to simply attend to the presented sequences.

In all combinations of modality, presentation and domain, we examined the effects of three different training types. All conditions presented the same set of sequences; *small* and *big* were not defined in absolute terms, they refer to the relative length of sentences within the same training set. In the *random* conditions, sequences of different length were presented in a random order; the *starting small* conditions involved incremental presentation of sentences ordered by length, starting with the shortest sequence; the *starting big* condition was the reverse of the *starting small* condition, starting with the longest sequences and gradually proceeding towards the shortest ones. It is important to point out that the shortest strings were not full sentences of the grammar, but they were structural units (phrases) of the language.

In the two-alternative forced choice (2AFC) test phase, participants were told that the sequences presented before were in an unknown language, and then they were presented with 24 sequence pairs of a grammatical sentence and a sentence containing a violation in each of the 24 trials. The grammatical-ungrammatical order within the sequence pair was counterbalanced across the trials. The order of the trials was random, but sentence-pairs were preset. Participants were instructed to choose the one which was more similar to sentences of the unknown language in the training phase and indicate it by pressing the corresponding key (‘1’ for the first sentence and ‘2’ for the second). The two sentences followed each other with 2000 ms pauses. Higher than chance scores (choosing the grammatical member of the pair significantly more than 50% of the time) was taken as evidence of learning.

## Results

Data were analyzed and visualized using IBM SPSS Statistics 20, JASP version 0.15.0.0^78^ and the R package ggplot2, version 3.3.5^44^. Descriptive statistics of accuracies in the 2AFC task are displayed in Table 1.

**Table 1:** Descriptive statistics of groups in different modality, presentation, domain and training conditions.

Modality	Presentation type	Domain	Training type	Mean accuracy (SD)	Age in years (SD)	Females/males
Auditory	Serial	Nonlinguistic	Random	0.58 (0.11)**	21.16 (2.24)	13/7
			Starting small	0.58 (0.09)***	20.66 (1.37)	13/7
			Starting big	0.56 (0.11)*	20.42 (1.47)	19/1
		Linguistic	Random	0.68 (0.10)***	22.13 (2.30)	15/5
			Starting small	0.75 (0.13)***	21.92 (1.90)	17/3
			Starting big	0.71 (0.12)***	21.91 (2.92)	15/5
Visual	Serial	Nonlinguistic	Random	0.59 (0.13)**	26.45 (7.35)	12/8
			Starting small	0.53 (0.13)	23.69 (2.45)	12/8
			Starting big	0.65 (0.16)***	21.68 (2.53)	16/4
		Linguistic	Random	0.58 (0.13)*	24.82 (7.74)	12/8
			Starting small	0.51 (0.12)	24.46 (2.21)	13/7
			Starting big	0.59 (0.17)*	22.38 (1.91)	15/5
Visual	Simultaneous	Nonlinguistic	Random	0.68 (0.16)***	20.91 (1.19)	13/7
			Starting small	0.70 (0.11)***	21.28 (0.95)	16/4
			Starting big	0.62 (0.14)***	20.59 (1.07)	18/2
		Linguistic	Random	0.66 (0.13)***	20.81 (2.19)	16/4
			Starting small	0.65 (0.13)***	22.96 (3.95)	11/9
			Starting big	0.62 (0.14)**	26.04 (5.90)	9/11

Descriptive statistics of the 2AFC accuracy task in groups in different Modality, Presentation Type, Domain and Training Type conditions. Differences from chance level (0.5) are calculated with one-sample t-tests, *: p < 0.05, **: p < 0.01, ***: p < 0.001. Mean age and proportion of females and males are also displayed for each group.

Post-hoc calculations of power estimates are included in Appendix 1. Detailed analyses of performance as a function of statistical regularities and violation types, as well as trial level performances were also conducted. These analyses showed that (1) participants performed above chance level in all violation types, (2) there were significant differences between performance by different violation types, (3) higher accuracy in violation types tended to co-occur with higher discriminability between the grammatical versus ungrammatical sequence based on of statistical features, and (4) statistical feature differences between grammatical and ungrammatical test items influenced accuracy on the word level, but not on the category level. These additional analyses are included in the supplementary materials.

**Figure 4:**
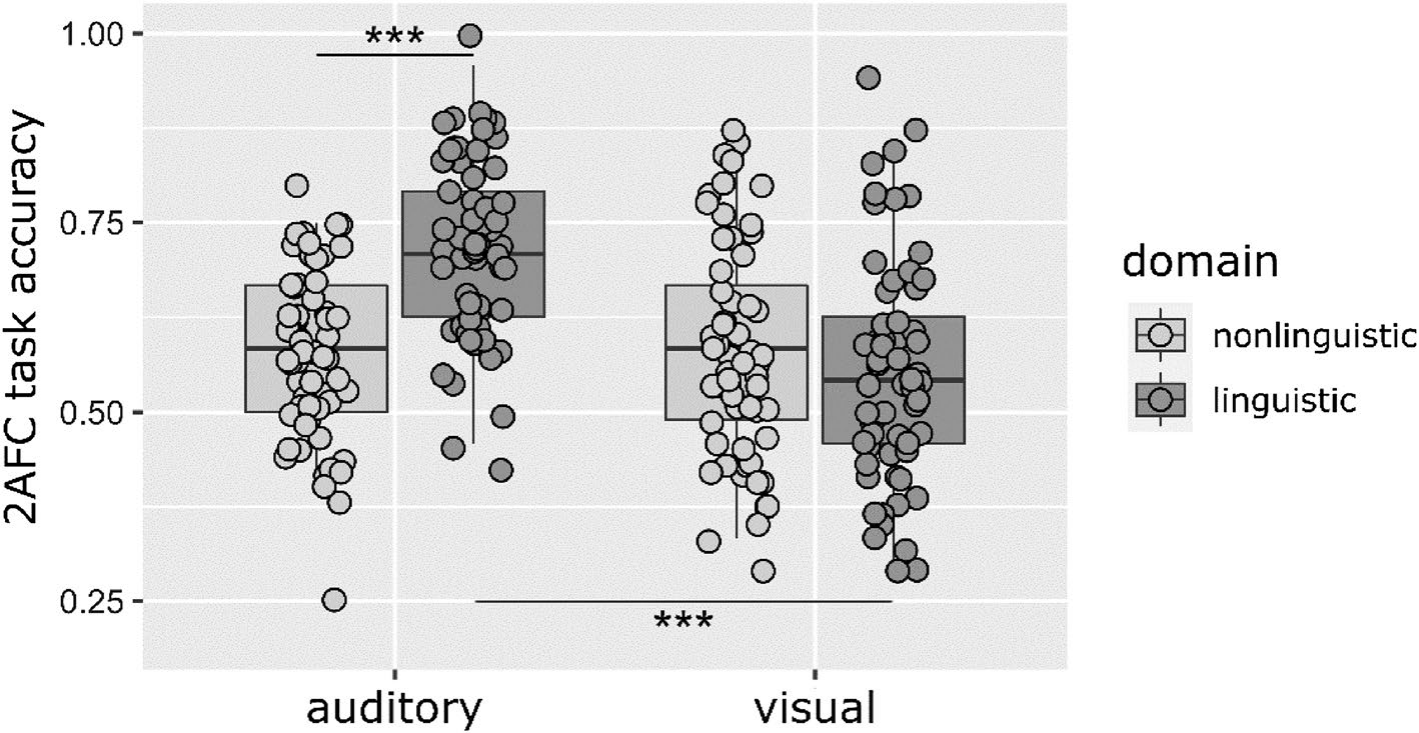
2AFC accuracy by Modality and Domain with *serial* presentation. Dots represent the mean accuracies of individual participants in a given group (a small amount of jitter was added to increase visibility). Lines in boxes represent group medians, box lengths illustrate the group interquartile range, and whiskers show minimum and maximum values. Outliers are participant data outside the 1.5 interquartile range beyond the first and third quartiles. *: *p* < 0.05, **: *p* < 0.01, ***: *p* < 0.001. Performance in the *auditory linguistic* condition was significantly better than performance in the *auditory nonlinguistic* condition and performance in the *visual linguistic* condition (regardless of Presentation Type).

First, we analyzed results for all conditions with *serial* presentation of stimuli. We conducted a three-way ANOVA to test the effects of Modality, Domain and Training Type. The effect of Modality was significant, F(1,228) = 19.12, p < 0.001, η_p_^2^ = 0.08, BF_10_ = 5.557e+6, performance in the *auditory* modality was higher than in the *visual* modality. The effect of Domain was also significant, F(1,228) = 11.83, p = 0.001, η_p_^2^ = 0.05, BF_10_ = 186,759.88, showing that participants performed better in conditions in the *linguistic* than in the *nonlinguistic* domain. The effect of Training Type was not significant, F(2,228) = 1.51, p = 0.223, η_p_^2^ = 0.01, BF_10_ = 0.914. The interaction of Modality*Domain was significant, F(1,228) = 26.83, p < 0.001, η_p_^2^ = 0.11, BF_10_ = 54,417.28 (Fig. 4). Post hoc analyses showed that in the *auditory* modality, performance in the *linguistic* domain was significantly higher than in the *nonlinguistic* domain, t(118) = -6.95, p < 0.001, r = 0.54, BF_10_ = 3.761e+7. In the *visual* modality, the difference between the two domains was not significant, t(118) = 1.08, p = 0.284, r = 0.10, BF_10_ = 0.33. In the *nonlinguistic* domain, Modality did not affect performance (t(118) = 0.57, p = 0.570, r = 0.05, BF_10_ = 0.23), while in the *linguistic* domain, the efficiency of *auditory* and *visual* learning differed significantly, with higher scores in the *auditory linguistic* than in the *visual linguistic* condition, t(118) = 6.52, p < 0.001, r = 0.51, BF_10_ = 4.910e+6.

**Figure 5:**
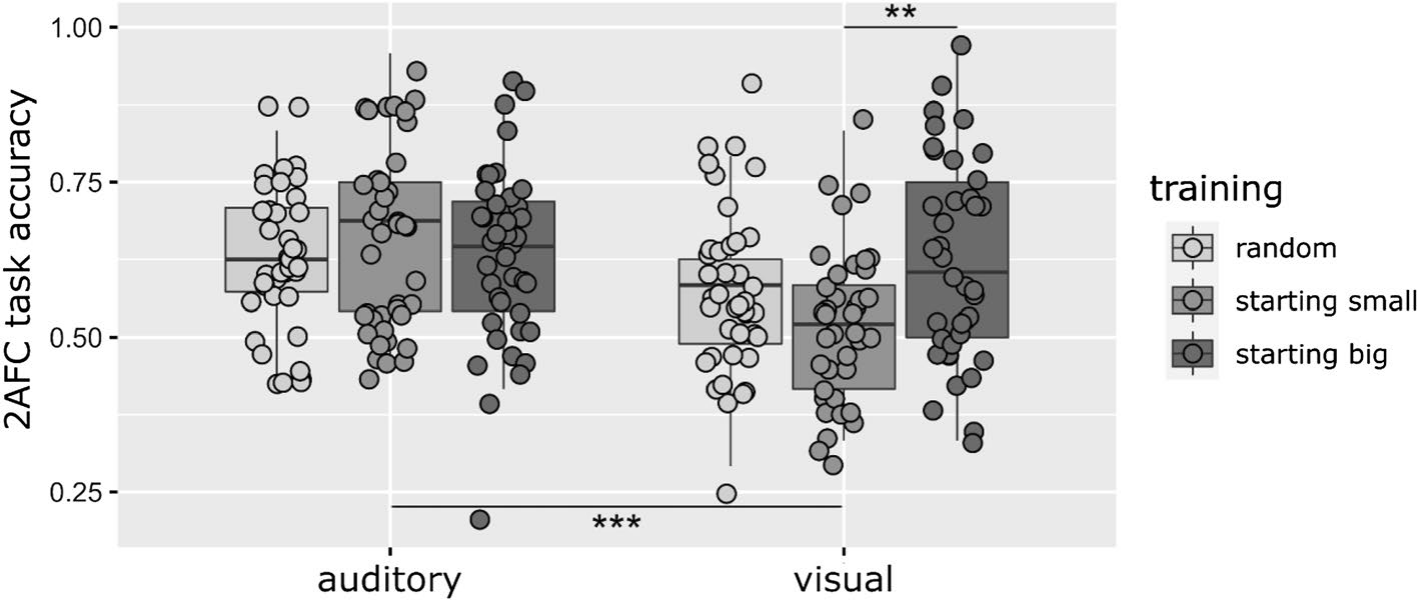
2AFC accuracy in the Modality*Training Type interaction in the case of *serial* presentation. Dots represent the mean accuracies of individual participants in a given group (a small amount of jitter was added to increase visibility). Lines in boxes represent group medians, box lengths illustrate the group interquartile range, and whiskers show minimum and maximum values. Outliers are participant data which are outside the 1.5 interquartile range beyond the first and third quartiles. *: *p* < 0.05, **: *p* < 0.01, ***: *p* < 0.001. Performance was significantly lower in the *visual starting small* condition than performance in the *auditory starting small* condition and performance in the *visual starting big* condition (regardless of Domain).

The interaction of Modality*Training Type was also significant, F(2,228) = 6.06, p = 0.003, η_p_^2^ = 0.05, BF_10_ = 4.59 (Fig. 5). Post hoc tests showed that in the *auditory* modality, Training Type has no significant effect, F(2,117) = 0.99, p = 0.374, η_p_^2^ = 0.02, BF_10_ = 0.18. However, in the *visual* modality, the effect of Training Type was significant, F(2,117) = 5.32, p = 0.006, η_p_^2^ = 0.08, BF_10_ = 6.07. Tukey pairwise comparisons showed that only the *starting small* and *starting big* groups were different (with higher performance in the *starting small* than in the *starting big* condition), p = 0.005, BF_10_ = 13.47, the *random* and *starting small*, and the *random* and *starting big* groups did not differ from each other, p = 0.110, BF_10_ = 1.96 and p = 0.459, BF_10_ = 0.41, respectively. In the case of the latter two comparisons, Bayes factors did not show evidence for equal performance in different training types. Further analyses showed that learning in the *auditory* modality was more efficient than in the *visual* modality in the case of the *starting small* condition, t(78) = 5.06, p < 0.001, r = 0.50, BF_10_ = 5497.80. The two modalities did not differ in the *random* and the *starting big* groups, t(78) = 1.74, p = 0.085, r = 0.19, BF_10_ = 0.86, t(78) = 0.50, p = 0.621, r = 0.06, BF_10_ = 0.26. However, in the former comparison, the Bayesian analysis did not show evidence for equal performance in the two modalities. No other interactions were significant.

**Figure 6:**
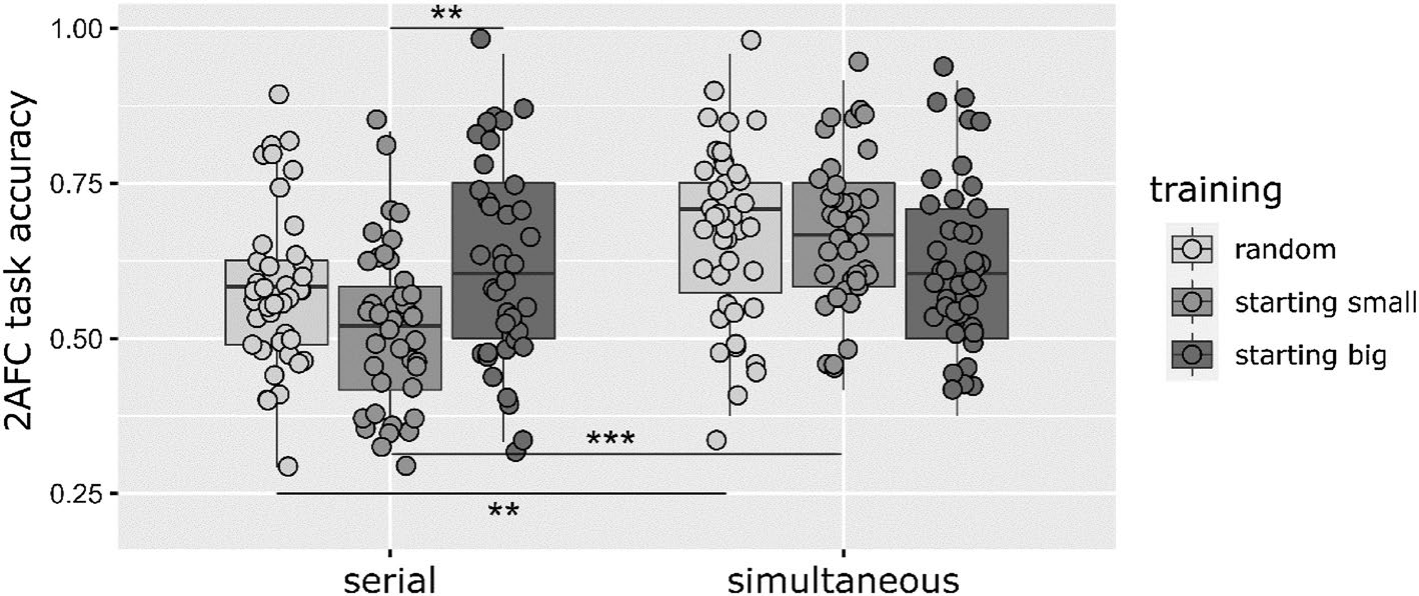
2AFC accuracy in the Presentation*Training Type interaction in the case of *visual* stimuli. Dots represent the mean accuracies of individual participants in a given group (a small amount of jitter was added to increase visibility). Lines in boxes represent group medians, box lengths illustrate the group interquartile range, and whiskers show minimum and maximum values. Outliers are participant data which are outside the 1.5 interquartile range beyond the first and third quartiles. *: *p* < 0.05, **: *p* < 0.01, ***: *p* < 0.001. *Serial starting small* performance was lower than *serial starting big* performance, and *simultaneous starting small* performance; and *simultaneous random* performance was higher than *serial random* performance (regardless of Domain).

We performed a second three-way ANOVA to test the effects of Presentation Type, Domain and Training Type on accuracy in the *visual* conditions. The effect of Presentation Type was significant, F(1,228) = 20.38, p < 0.001, η_p_^2^ = 0.08, BF_10_ = 1092.94; performance was higher with *simultaneous* presentation than with *serial* presentation. The main effects of Domain and Training Type were not significant, F(1,228) = 2.11, p = 0.148, η_p_^2^ = 0.01, BF_10_ = 0.18, and F(2,228) = 0.10, p = 0.371, η_p_^2^ = 0.01, BF_10_ = 0.56, respectively. The interaction of Presentation Type*Training Type was significant, F(2,228) = 6.10, p = 0.003, η_p_^2^ = 0.05, BF_10_ = 2.86 (Fig. 6). Post hoc analyses showed that in the case of *serial* presentation, the effect of Training Type was significant, F(2,117) = 5.32, p = 0.006, η_p_^2^ = 0.08, BF_10_ = 6.07; Tukey pairwise comparisons showed that *starting small* was less efficient than *starting big* training, p = 0.005, BF_10_ = 13.47, but learning with *random* and *starting small*, and *random* and *starting big* training did not differ from each other, p = 0.110, BF_10_ = 1.96 and p = 0.459, BF_10_ = 0.41, respectively. (Note that this analysis is the same as the post hoc analysis of Training Type in the case of the *visual* modality in the previous ANOVA.) The effect of Training Type was not significant in the case of *simultaneous* presentation, F(2,117) = 1.75, p = 0.178, η_p_^2^ = 0.03, BF_10_ = 0.33. When analyzing the effect of Presentation Type in each Training Type, we found that in the case of *random* and *starting small* training, *simultaneous* presentation resulted in higher performance levels than *serial* presentation, t(78) = -2.77, p = 0.007, r = 0.30, BF_10_ = 5.99, and t(78) = -5.58, p < 0.001, r = 0.53, BF_10_ = 37,631.56. In the case of *starting big* training, there was no difference between presentation types, t(78) = -0.06, p = 0.951, r = 0.01, BF_10_ = 0.23. No other interactions were significant.

**Figure 7:**
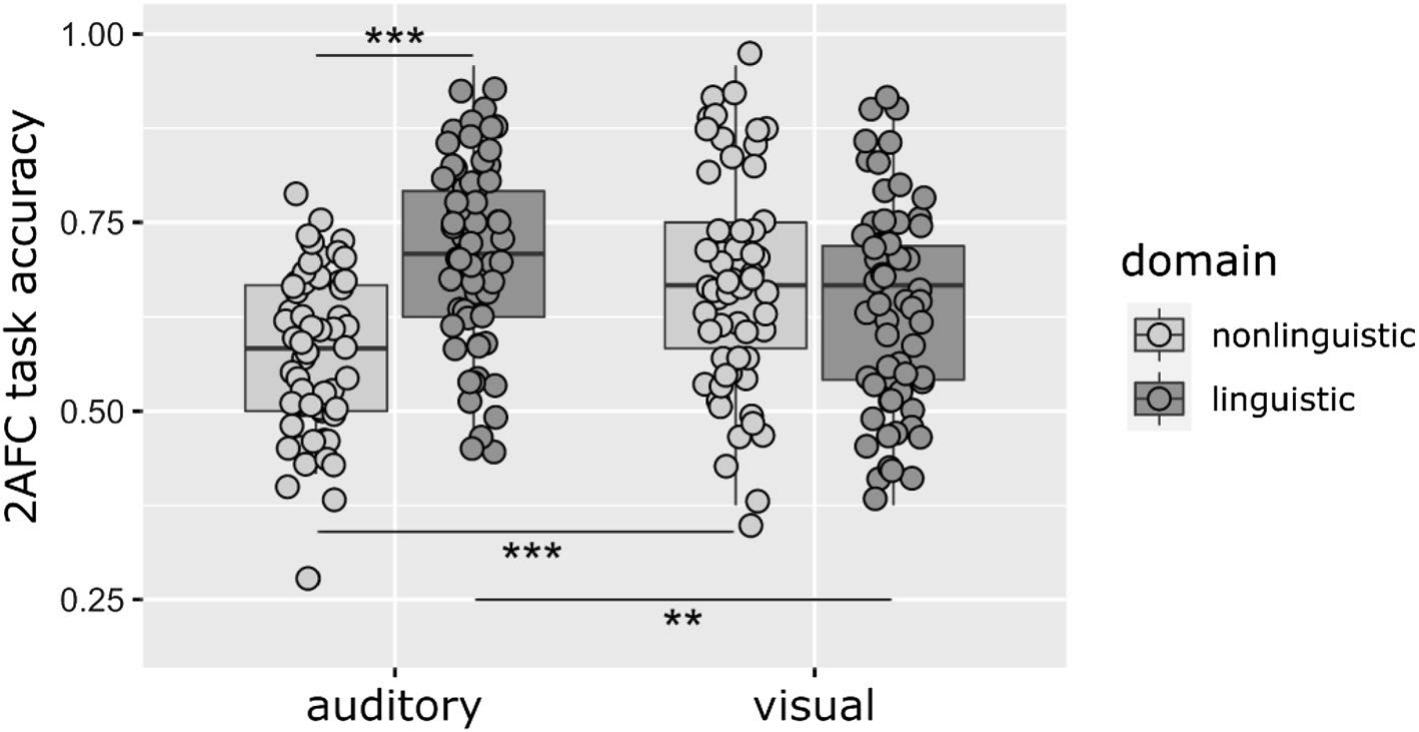
2AFC accuracy by Modality and Domain with the optimal Presentation for each modality (*serial* presentation for *auditory* stimuli, and *simultaneous* presentation for *visual* stimuli). Dots represent the mean accuracies of individual participants in a given group (a small amount of jitter was added to increase visibility). Lines in boxes represent group medians, box lengths illustrate the group interquartile range, and whiskers show minimum and maximum values. Outliers are participant data which are outside the 1.5 interquartile range beyond the first and third quartiles. *: p < 0.05, **: p < 0.01, ***: p < 0.001. Performance in the *auditory linguistic* condition was higher than performance in the *auditory nonlinguistic* condition and performance in the *visual linguistic* condition; and performance in the *visual nonlinguistic* condition was superior to performance in the *auditory nonlinguistic* condition (regardless of Training Type).

We performed a third three-way ANOVA to test the effects of Modality, Domain, and Training Type for optimal presentation for each modality, i.e., *serial* presentation for *auditory* stimuli, and *simultaneous* presentation for *visual* stimuli. With presentation type fitted to modality, the effect of Modality was not significant, F(1,228) = 0.34, p = 0.560, η_p_^2^ < 0.01, BF_10_ = 1335.81. On the other hand, the effect of Domain was significant, F(1,228) = 13.34, p < 0.001, η_p_^2^ = 0.06, BF_10_ = 46,289.46, showing that participants performed better in conditions in the *linguistic* domain than in the *nonlinguistic* domain. The effect of Training Type was not significant, F(2,228) = 2.30, p = 0.102, η_p_^2^ = 0.02, BF_10_ = 0.17. The interaction of Modality*Domain was significant, F(1,228) = 26.24, p < 0.001, η_p_^2^ = 0.10, BF_10_ = 6922.99. This interaction is illustrated in Fig. 7. Post hoc analyses showed that in the *auditory* modality, performance in the *nonlinguistic* domain was significantly lower than in the *linguistic* domain, t(118) = 6.95, p < 0.001, r = 0.49, BF_10_ = 3.761e+7. In the *visual* modality, the difference between the two domains was not significant, t(118) = 0.95, p = 0.346, r = 0.09, BF_10_ = 0.291. In the *nonlinguistic* domain, the performance in the *visual* modality was higher than in the *auditory* modality, t(118) = 4.03, p < 0.001, r = 0.35, BF_10_ = 216.78, and in the *linguistic* domain, we observed the opposite pattern, with significantly higher performance in the *auditory* than in the *visual* modality, t(118) = 3.21, p = 0.002, r = 0.28, BF_10_ = 17.97. No other interactions were significant.

As pointed out by one of the reviewers of the original manuscript, in the *auditory nonlinguistic* conditions, the use of musical tones may give rise to musical features like contours (the ascending and descending pattern between tones) and intervals (the relative pitch change between tones)^75^. Taking this into account, a possible explanation for lower performance in the *auditory nonlinguistic* condition is that statistical patterns of these emergent musical features might conflict with statistical information imposed by the grammar alone. To check this possibility, we compared the main effect of Domain and the Modality*Domain interaction in test trials where grammatical and musical patterns converged (based on the statistics of the learning phase) and in test trials where grammatical and musical statistics diverged in supporting a choice between the grammatical versus ungrammatical item. We found that the main effect of Domain and the Modality*Domain interaction was more prominent in test trials where emergent musical patterns and grammatical patterns did not converge. A thorough description of the recoding process and the analysis is provided in Appendix 2.

Since age was not entirely balanced between groups, we checked whether it affected learning to control for potential biases. Age had a very weak, but significant negative relationship with performance, r(356) = − 0.11, p = 0.039, however, the Bayesian analysis did not show evidence for this relationship, BF_10_ = 0.64. To examine a potential effect on the results of ANOVA analyses, we performed three further ANCOVAs with Age as a covariate. When testing the effects of Modality, Domain and Training Type in the case of *serial* presentation, we found that Age had no significant effect on performance, F(1,226) = 0.07, p = 0.790, η_p_^2^ < 0.01, BF_10_ = 0.17. Similarly, when testing the effect Presentation Type, Domain and Training Type in the case of *visual* stimuli, Age had no significant effect, F(1,226) = 0.17, p = 0.677, η_p_^2^ = 0.01, BF_10_ = 0.16. When testing the effect of Modality, Domain, and Training Type in the case of optimal presentation type, Age was a significant covariate, F(1,225) = 6.02, p = 0.015, η_p_^2^ = 0.03, BF_10_ = 8.01, but including it as a covariant did not change the pattern of findings: the effects of Domain and Modality*Domain remained significant.

## Summary and discussion

Statistical learning operates across many different areas of cognition on different stimuli, but the effects of modality, presentation, domain and training type together with the interaction of these factors have not been examined together and systematically in the statistical learning literature. To fill this gap, we investigated the effects of these factors in an artificial grammar task. When stimuli were presented serially, learning was more effective in the auditory than in the visual modality. This modality effect was particularly pronounced in the linguistic domain. With simultaneous presentation of visual stimuli, the auditory advantage over the visual modality disappeared. A significant domain effect showed that learning linguistic patterns results in higher performance than learning nonlinguistic patterns. However, the linguistic advantage over learning the nonlinguistic material was only present in the auditory modality. The auditory linguistic condition had an overall advantage over other modality-domain types. Training type did not have any general effect on the acquisition of the grammar, but starting big enhanced performance in the case of serial visual presentation relative to starting small training, and starting small training with serial visual materials resulted in lower performance than starting small training with simultaneous visual materials. The results and their implications are discussed in the context of earlier findings and in more detail in the following sections.

### Effects of modality and presentation

We expected an auditory advantage in statistical learning with serial presentation of stimuli. This assumption was supported by our results: the grammar was easier to learn in the auditory than in the visual modality, in line with previous results by Conway and Christiansen^3^, who found the same pattern with a simpler grammar. These observations suggest that regardless of the complexity and structure of the pattern to be learned, when stimuli are presented serially, learning is more effective in the auditory than in the visual modality. Such modality effects in statistical learning tasks might reflect differences in general information processing mechanisms in sensory modalities. Supporting this notion, Conway and Christiansen^30^ demonstrated that well-known primacy and recency effects in serial recall^45,46^ are also present in statistical learning. Moreover, the advantage of auditory over visual presentation, demonstrated in previous studies and in the current one, had also been described outside the field of statistical learning, for instance, in the memory for order of words in sequences in a word list recall task^47^.

However, with presentation type optimized, i.e., when items of the sequence are presented serially in the auditory, and simultaneously in the visual modality, the auditory advantage disappeared and learning was equally efficient in both modalities, in concert with previous results of Conway and Christiansen^30^. The findings of Saffran^31^ also provide indirect support for this claim, however, she only found an advantage of simultaneous over serial presentation for visual stimuli with the same predictive grammar we also used (but not with the nonpredictive grammar). As she discusses, it is unclear whether this pattern was due to the advantage of visual simultaneous learning for the predictive grammar, or the disadvantage for the non-predictive grammar. Taken together, (1) our results support the advantage of auditory over visual statistical learning with serial presentation; (2) simultaneous presentation seems to benefit visual statistical learning of sequences over visual serial presentation; and (3) when presentation is optimized for modality, there is no difference between modalities in learning efficiency.

The advantage of simultaneous compared to serial visual presentation raises the possibility that modality effects might be specific to or at least interact with structure type. In statistical learning, modality effects are generally investigated with sequential structures (with some exceptions^48^). However, while auditory perception and processing seems to be suited for processing temporal information, which is inherently sequential, vision is better suited to processing spatial than temporal information, which can be both sequential and nonsequential (as concluded by Freides^49^, and Conway and Christiansen^3^, but see also other studies^48,50–52^). Testing modality effects can be challenging in the case of nonsequential structures, although not impossible^48^, due to the sequential organization of most types of auditory information. As this modality effect might be limited to sequential processing, further studies should target nonsequential structures to broaden our knowledge about modality effects in statistical learning and other domains of cognition. To conclude, the present study (1) confirms modality effects observed in earlier studies and extends them to predictive dependencies and a category-based grammar, (2) shows that these modality effects can be structure dependent.

### Domain effects

Based on previous findings^31^, we expected no advantage of learning the grammar with linguistic over nonlinguistic stimuli (although see^70^ for results with a linguistic advantage with a different design). This assumption was only partially supported by our findings. In the case of serial presentation, performance was higher in linguistic than in nonlinguistic conditions. We observed a similar domain effect in the analysis including serial auditory and simultaneous visual learning (i.e., the optimal presentation for each modality). A possible explanation for a linguistic advantage would be that the grammar was explicitly created to mimic predictive dependencies and word categories common in human languages: in the original design, Saffran^31^ argued that learning constraints should be tailored to the stimuli for effective learning, thus, different constraints might be advantageous for learning linguistic and nonlinguistic stimuli, as different chunking and grouping mechanisms might operate in these domains (e.g., different constraints for linguistic structures versus musical structure in the auditory modality, and for symbol sequences versus complex real-life visual scenes in the visual domain). This type of structure with predictive dependencies and word categories, characteristic of language, might be optimal for learning linguistic materials. A further potential explanation of the linguistic over nonlinguistic advantage is that participants, although they are not instructed to do so, might also apply explicit memorization strategies for linguistic materials (e.g., rehearsal of sequences) which are less available for other types of stimuli.

However, the presence of the domain effect in the auditory conditions draws attention to the potential influence of stimulus specific factors beyond general effects in statistical learning. In the auditory nonlinguistic condition, the use of musical tones may give rise to musical features like contours (the ascending and descending pattern between tones) and intervals (the relative pitch change between tones)^75^, which might support or be in conflict with grammatical information. Indeed, the linguistic advantage observed in the auditory modality was challenged by further analyses suggesting that lower performance in the nonlinguistic condition might have been caused by conflicting grammatical and musical patterns. Therefore, also in line with the result of no linguistic advantage in the visual modality, our results do not support *general* domain effects in statistical learning: the efficiency of learning may depend on more stimulus-specific features. Stimulus- and task specific learning effects are not surprising, since statistical information is not the only cue to finding structure in environmental stimuli. In cases of contradicting cues, other sources of information may override it (see e.g. prosody over statistics: Johnson and Seidl^73^; familiar units over statistical cues: Poulin and colleagues^74^), although in other cases, learners may rely on statistical features over other information types (statistical cues over similarity: Tillman and McAdams^79^).

To summarize, we found an advantage of statistical learning in auditory linguistic conditions compared to all other conditions, including visual linguistic learning. In addition, performance in the auditory nonlinguistic condition was weaker than in other conditions. These results show that the effectiveness of statistical learning may be influenced by the domain of learning (e.g. linguistic versus nonlinguistic). However, in our study this domain effect was confounded with other emergent patterns in the stimuli: musical patterns (contours and intervals) in tone sequences were in conflict with statistical patterns defined by the grammar, making learning in the auditory nonlinguistic conditions more difficult than learning sequences of syllables. Further studies are needed to clarify the nature of and control for such effects and their interaction with domain and modality. These results suggest that instead of global domain effects, stimulus specific effects shape statistical learning which may also depend on task type, design and features of the learning material.

### Training effects

To examine the influence of input characteristics on statistical learning, we also explored training effects across different modalities and domains. We hypothesized that starting small would facilitate the acquisition of the category based grammar through enabling the generation of simple and flexible hypotheses about the underlying rules. In contrast, we expected starting big to yield lower learning performance due to less effective hypothesis generation. However, we only found an effect of training with serial presentation in the visual modality: here, regardless of stimulus domain (i.e. both in the linguistic and nonlinguistic conditions), starting small training had an adverse effect on performance, while starting big training facilitated learning. This pattern of results for different training types suggests that the way of stimulus presentation can affect statistical learning in important and perhaps modality and domain-dependent ways. The visual processing system seems to be optimized for spatial rather than temporal processing^49^, and the starting big presentation might compensate for the insufficient availability of information in the serial presentation.

The above pattern of results is in contrast with earlier findings about the starting small effect in visual statistical learning showing enhanced acquisition of structure with starting small training and simultaneous presentation in the visual modality both in the linguistic^53^ and the nonlinguistic domain^35^. These contradictory findings may be explained by differences in the grammars: previous studies applied recursive grammars in which the structure was based on the non-adjacent combination of item pairs. Thus, the initial acquisition of adjacent pairs of these legal combinations is essential, and increasingly more difficult when embedded in longer sequences: the complexity (the number of different sequences the grammar can generate) of recursive grammar sentences exponentially increases as a function of length^53^. Starting small training targets this problem with presenting pairs with just the two adjacent items in the beginning. However, the grammar that we used is different in structure. Here, complexity does not increase with sentence length as much as in the case of recursive grammars. Poletiek and colleagues^53^ argued that the key to the starting small effect is the presentation of less complex, and not necessarily shorter, sequences. As a result, the acquisition of this type of grammar may not profit as much from starting small training. However, statistical properties of ‘small’ phrases were not controlled for, and post-hoc analyses of these regularities do not show systematic differences. Shorter sequences with less complex statistical regularities than the longer ones might yield larger benefits in starting small: this would be a design worthy of implementation in a future study.

A further reason for the absence of the starting small effect might be that shorter sequences induce explicit rule search strategies which decrease the efficiency of learning complex statistical patterns^71,72^. It is also possible that we have not provided sufficient information in the beginning of training in the starting small presentation conditions of our study for beneficial effects. Given the variability of items within phrases, the training might have been too short for participants to acquire these basic units and a longer training with ‘small’ phrases might have resulted in stronger or more explicit representations, which might have then served better as building blocks in later parts of the training with more complex material. To summarize, as training effects might significantly depend on grammar or structure type, further studies are needed to determine their scope. A larger sample size would also benefit exploring training effects further, as post-hoc comparisons in the Modality*Training Type interaction were not powered enough to unequivocally show either the presence or the absence of a difference.

### Considerations about pattern and stimuli characteristics

Statistical learning is an umbrella term covering the acquisition of several types of patterns and systems, for instance, segmenting words from a speech stream^9,11,70^, learning regularities in real-world scenes^27^, spatial locations^13,14^, acquiring visual patterns and faces^6,7,28,54^, or learning musical systems^87,88^. Even in the case of learning sequential information in artificial grammar learning tasks, the structure to be acquired is highly variable: phrasal^31^, finite-state^55^, central-embedded or right-branching recursive^35,38^, and non-adjacent dependency grammars^56^ are all applied. The literature on the effects of modality^3^, presentation^30,31^, domain^31,70^ and training effects^25–32,32–39,41,42^, and more broadly, input properties e.g.,^79,83–86^ also relies on results from statistical learning studies working with a large variety of structure types. Although we used a category-based artificial grammar consisting of predictive dependencies in the present study, we aimed to explore domain, modality and training effects on statistical learning in general. Our results extend and confirm previous findings from different tasks and stimulus sets on modality, domain, presentation and training effects in statistical learning. At the same time, contradicting findings from tasks with different statistical structure types (e.g., while Saffran^31^ found no linguistic advantage in an artificial grammar task, Hoch, Tyler and Tillmann^70^ found that learning linguistic materials was more successful than learning nonlinguistic materials in a segmentation paradigm) draw the attention to a possible interaction of input characteristics and structure type, which should be addressed by future studies.

Beside structure type, stimulus type is also an underexamined, yet significant factor in statistical learning^57^. Linguistic stimuli can take many forms in different modalities and different constraints may apply in learning from speech streams versus written texts versus gesture sequences. On the other hand, in the nonlinguistic domain, various types of musical and environmental sounds can be used as auditory stimuli, while for the visual modality, applied stimuli range from colorful squares through spatial locations to complex symbols, all organized by potentially different statistical constraints. The constraints for optimal acquisition might be specific not only to modality and/or domain, but to stimulus type as well. Previous results also suggest that learning efficiency for different stimulus types interacts with age, as well: Raviv and Arnon^67^ and Shufaniya and Arnon^68^ found different developmental trajectories during childhood for different stimulus types in statistical learning. Further studies should explore such specificity in statistical learning: investigating modality, domain and training effects with a diverse set of structure and stimulus types in different ages is an important future direction.

### Methodological and psychometric limitations

One of the limitations of our study is a general methodological problem that many statistical learning studies face: we only measured learning offline, that is, after the learning phase. This post hoc measurement is problematic from multiple aspects. First, this way, we cannot gain information about the process and dynamics of learning. Second, as a consequence, we measure knowledge only at retrieval, which is a different process from encoding (for a discussion of implications for statistical learning, see^58^). This is especially important when modality- and domain specific effects are in the focus, as their encoding and retrieval processes might differ^59,60^. Third, the typically applied offline forced-choice tests recruit cognitive abilities distinct from statistical learning, for instance, decision-making and working memory processes^61,62^. Individual variations in these abilities might also make the measurement of statistical learning noisy and unreliable. A potential solution to these pitfalls is relying on online measurements: for instance, measuring reaction times to one or more predictable items during the training allows to infer changes in the efficiency of processing and predicting items in the pattern. This can be then applied as a measure of statistical learning^13,14,62–65^.

There are also psychometric aspects to be considered in future testing. Offline forced choice tasks often apply a relatively low number of trials. However, in a task type where group performance is just slightly different from the chance level most of the time, on the individual level, above-chance performance is difficult to distinguish from chance-level performance^66^. In our case, with a mean score of 0.62 yielded from the 24 trials in the two-alternative forced choice task, there is an 8% chance that an individual performed above chance merely by accident based on the binomial distribution. This is even more likely in conditions where mean performances were lower. (However, increasing the number of test trials, and thus participants’ exposure to ungrammatical sequences, may weaken or alter acquired statistical representations. This effect could be minimized by including ungrammatical trials without any systematic statistical biases or controlled for by applying statistical methods which include trial order as a random factor.) Including trials with systematically varying difficulty would also make a better targeted method, as participants with different levels of knowledge could be more accurately tested. Thus, increasing the number and variability of trials would make results less noisy and more reliable, resulting in a better statistical learning task.

Finally, it is also a limitation to be addressed by future studies that we did not collect any information on backgrounds in musical training for the participants. In the serial non-linguistic condition, tone sequences created short melodies, which participants with a musical training might have found easier to process. Since more general beneficial effects of musical training have been reported for memory and learning^80–82^, controlling for effects of musical training on performance would be relevant not just for the statistical learning of tone sequences, but for other modalities and domains as well.

## Conclusions

The present study demonstrates that the efficiency of the acquisition of statistical structures may show considerable differences depending on the specific modality, domain, and presentation type. Most importantly, our findings show the advantage of sequential learning of auditory linguistic stimuli over other modalities and domains. Moreover, when grammar-based and musical features were matched in the nonlinguistic auditory condition, similar levels of performance were reached as in the linguistic auditory condition. This indicates the presence of constraints in statistical learning: serial presentation with this type of sequential structure with predictive dependencies and abstract categories might be optimal for learning auditory stimuli, while other stimulus types might profit more from other structure varieties. Our results also suggest that optimal, that is, simultaneous presentation type can boost learning performance in the visual modality. However, we found no general training effect in the present study, which indicates that training effects might also depend on the specific structure to be acquired. Our findings show that learning is constrained by the modality and presentation type together with the specific stimulus characteristics of the input, and call for broadening the scope of research by testing input effects on statistical learning with a wider range of structure and stimulus types.

## Supplementary Information

Supplementary Information 1.

Supplementary Information 2.

Supplementary Information 3.

## Data Availability

Study materials and data of the experiment are available at https://osf.io/hzg7w/?view_only=639eb1b8b8dd4325a7a5ce25c08cee9b.
