# Supplementary material for: Modality, presentation, domain and training effects in statistical learning
Source: Sci Rep. 2022 Dec 3;12:20878. doi: 10.1038/s41598-022-24951-7 (PMC9719496; doi:10.1038/s41598-022-24951-7)
Supplement: Supplementary file 1 — Supplementary Information 1. [file 41598_2022_24951_MOESM1_ESM.docx]

**APPENDIX 1**

| Table 1. Power analyses of the ANOVAs in the study | | | | | | |
| --- | --- | --- | --- | --- | --- | --- |
| factor | ANOVA 1 | | ANOVA 2 | | ANOVA 3 | |
|  | significance | power | significance | power | significance | power |
| modality | *** | .992 | NA |  |  | .098 |
| presentation type | NA | NA | *** | .994 | NA | NA |
| domain | *** | .928 |  | .304 | *** | .953 |
| training type |  | .319 |  | .222 |  | .464 |
| modality * domain | *** | .999 | NA |  | *** | .999 |
| modality * training type | ** | .882 | NA |  |  | .203 |
| presentation type * domain | NA | NA |  | .052 | NA | NA |
| presentation type * training type | NA | NA | ** | .884 | NA | NA |
| domain * training type |  | .091 |  | .069 |  | .107 |
| modality * domain * training type |  | .180 | NA | NA |  | .179 |
| presentation type * domain * training type | NA | NA |  | .129 | NA | NA |

Note: ANOVA 1: modality, domain and training effects with serial presentation on stimuli, ANOVA 2: presentation type, domain and training effects with visual stimuli, ANOVA 3: modality, domain and training effects with optimal presentation type for each modality, *: p <.05, **: p < .01, ***: p < .001
