# Supplementary material for: Modality, presentation, domain and training effects in statistical learning
Source: Sci Rep. 2022 Dec 3;12:20878. doi: 10.1038/s41598-022-24951-7 (PMC9719496; doi:10.1038/s41598-022-24951-7)
Supplement: Supplementary file 2 — Supplementary Information 2. [file 41598_2022_24951_MOESM2_ESM.docx]

**APPENDIX 2**

In the acoustic nonlinguistic condition stimulus sequences may have yielded emergent perceptually relevant musical features influencing learning, which the original analysis did not take into account. To control for the effect of these features, we did a post-hoc analysis of contours and intervals. The stimuli in this condition were 16 musical tones with the following frequencies recoded into numbers 1-16 where higher numbers represent higher pitch, and bigger differences in numbers correspond to bigger differences in perceived pitch differences:

**220Hz 240Hz 263Hz 287Hz 314Hz 343Hz 374Hz 409Hz 447Hz 488Hz 534Hz 583Hz 637Hz 696Hz 760Hz 831Hz 🡪 1 2 3 4 5 6 7 8 9 10 11 12 13 14 15 16**

**The syllables in the linguistic conditions were paired with tones (the syllable-frequency correspondences were arbitrary) and mapped onto the same numbers as their tone pairs**

**bif dők dup gal hep kav lam lor mib neb péf rász rud sot szig tez 🡪 220Hz 240Hz 263Hz 287Hz 314Hz 343Hz 374Hz 409Hz 447Hz 488Hz 534Hz 583Hz 637Hz 696Hz 760Hz 831Hz**

Thus, each sentence in each condition could be rewritten with numbers:

**mib szig tez dők lam 🡪 447Hz 761Hz 831Hz 240Hz 374Hz 🡪 9 15 16 2 7**

We recoded training and test sentences as contour and interval changes bigrams and trigrams. A contour is defined as the ascending or descending pattern of tones. An interval is defined as the number of relative pitch changes between tones. Thus, the previous sentence can be rewritten as a contour:

**UP UP DOWN UP**

And as a sequence of intervals:

**+6 +1 -14 +5**

Note that the recoded sequences are one item shorter than the original sequences because contours and intervals indicate relationships between two items. For this reason, contour and interval patterns for bigrams were one item long, and contour and interval patterns for trigrams were two items long. The 5 item sentence in the example has the following bigram contours: UP, UP, DOWN, UP; the following bigram intervals: +6, +1, -14, +5; the following trigram contours: UP-UP, UP-DOWN, DOWN-UP; and the following trigram intervals: +6-+1, +1--14, -14-+5.

We calculated bigram and trigram frequencies for the training material for the following features: category, word, contour and interval changes. This resulted in eight factors: *category bigram frequency*, *category trigram frequency*, *word bigram frequency*, *word trigram frequency*, *contour bigram frequency*, *contour trigram frequency*, *interval bigram frequency*, and *interval trigram frequency*. Then we calculated frequency differences for these measures for all test sequence pairs. For each measure, we subtracted the given feature frequency of the grammatical item from the feature frequency of the ungrammatical item. This resulted in a positive number if the frequency was higher in the grammatical test item, and a negative number if the frequency was higher in the ungrammatical test item. In a regression analysis, we tested which factors (frequency differences of category, word, contour, and interval between grammatical and ungrammatical test items) had a significant effect in shaping responses. We found that *word bigram frequency difference*, *category bigram frequency difference*, *contour trigram frequency difference*, *interval bigram frequency difference* and *interval trigram frequency difference* were significant factors in predicting responses in the test trials. As *word bigram frequency difference* and *category bigram frequency difference* were strongly correlated, these two factors show the same effect. Table 1 shows the correlations between the different factors. Table 2 shows the effects of the individual predictors in the regression analysis.


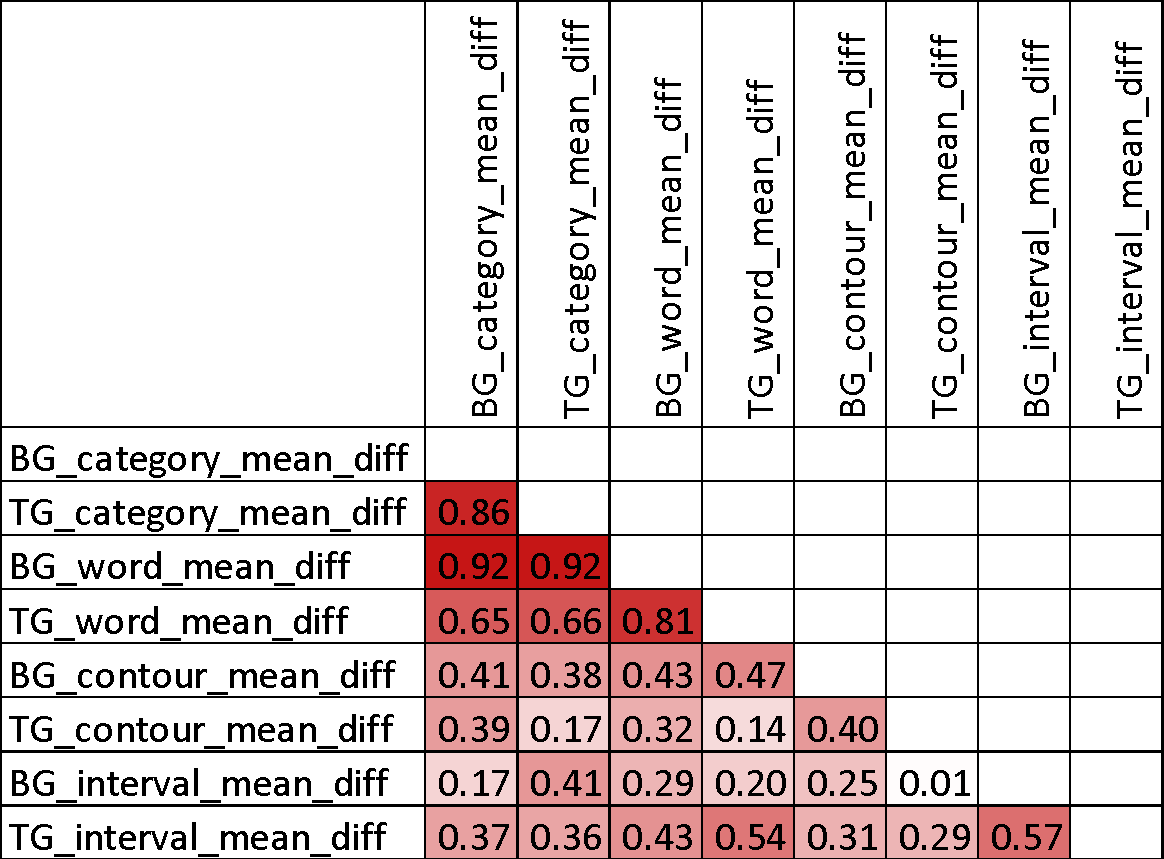


Table 1. Correlations between frequency difference measures in the test item pairs as calculated based on the training items. BG: bigram, TG: trigram


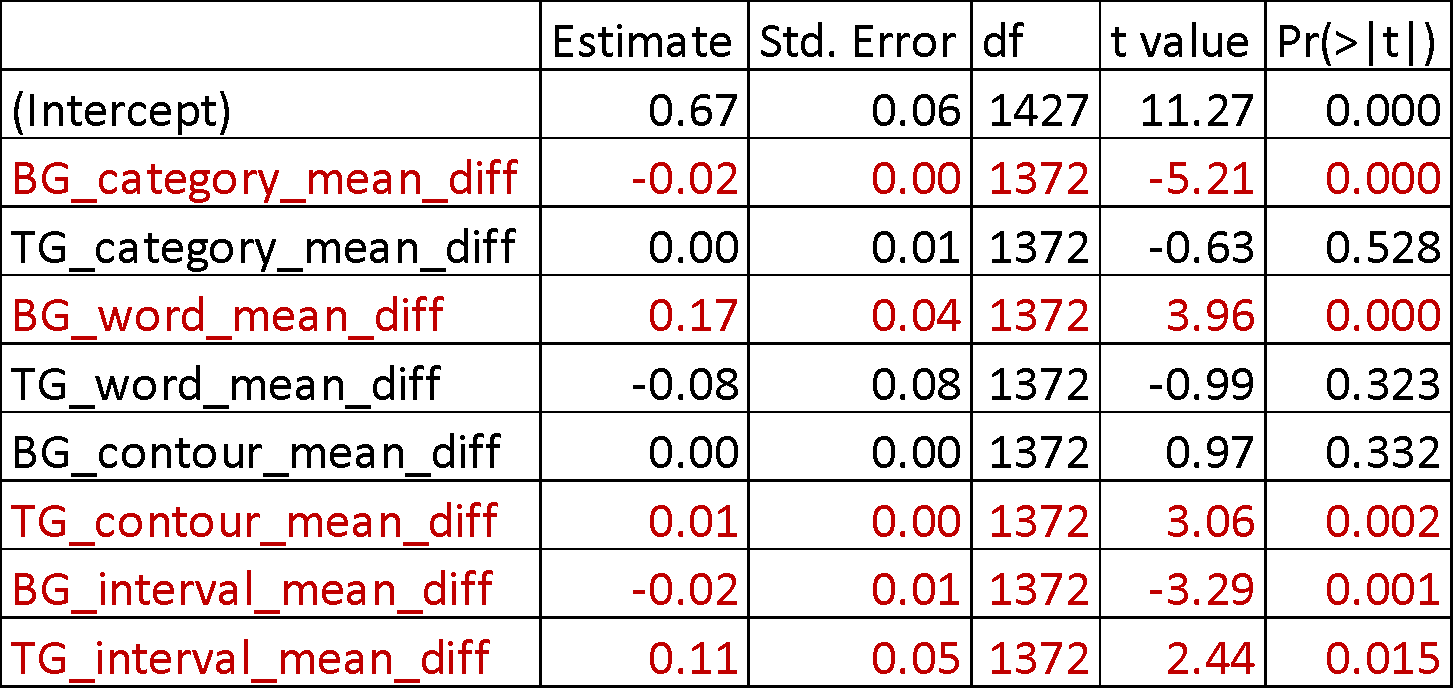


Table 2. Factor effects on responses in the test phase. BG: bigram, TG: trigram

To control for emergent musical features, we analyzed separately test trials where the grammatical versus ungrammatical items differed in both musical features and grammatical statistics (grammar-based and musical feature differences between items matching) and trials where there was no difference between the grammatical and ungrammatical item from the aspect of emergent musical features (existing grammar-based differences but no musical feature differences between grammatical and ungrammatical items). We analyzed the effect of Modality, Domain together with their interaction separately for the three musical features which were significant predictors (*contour trigram frequency difference*, *interval bigram frequency difference* and *interval trigram frequency difference*). Since they were only moderately correlated, and thus there was only partial overlap in trials where these three features had similar frequencies, we could increase trial number by analysing results separately for the three features. Table 3 shows the results of the analyses.


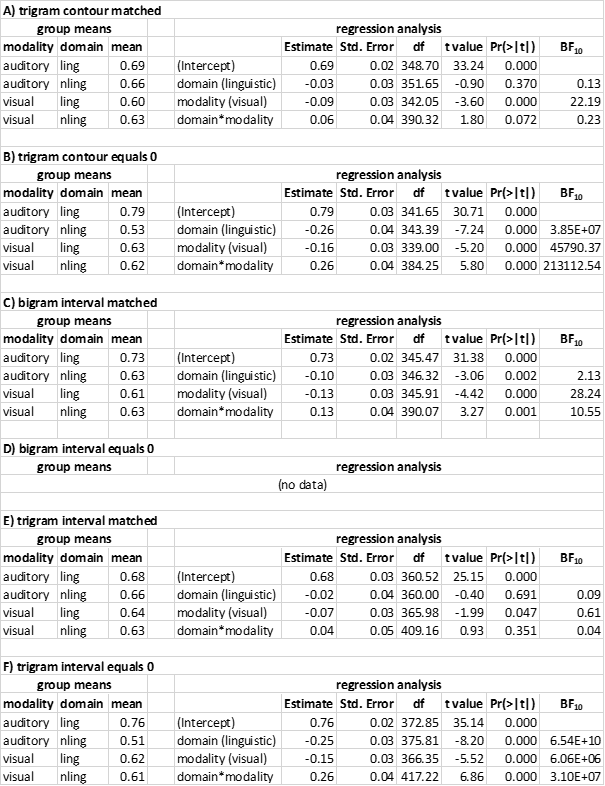


Table 3. Mean performance levels by conditions and results of regression analyses in different test trial subsets. Note that “matched” denotes that grammar-based and musical feature differences between grammatical and ungrammatical items are consistent with each other, and “musical feature equals 0” denotes that there were grammar-based differences but no musical feature differences between grammatical and ungrammatical items. A) trials where trigram contour differences were matched to differences in grammar-based statistics; B) trials where trigram contour difference was 0; C) trials where bigram interval differences were matched to differences in grammar-based statistics; D) trials where bigram interval difference was 0 (there were no such trials); E) trials where trigram interval difference was matched to differences in grammar-based statistic; F) trials where trigram interval difference was 0.

Compared to the original analyses, the results of these analyses show 1) smaller or no difference between linguistic and nonlinguistic conditions and smaller or no Modality*Domain interaction in the case of trials where grammar-based and musical feature differences were matched, and 2) more emphasized differences and Modality*Domain interaction where musical feature differences were 0. These results suggest that lower performance in the acoustic nonlinguistic condition compared to the acoustic linguistic condition was (at least, partly) accounted for by the fact that word-level and emergent musical features pointed in the opposite direction in some test trials in the acoustic nonlinguistic condition. This observation calls for further studies relying on different stimuli or controlling for such effects in acoustic nonlinguistic conditions.
